# Working Memory and Its Mediating Role on the Relationship of Math Anxiety and Math Performance: A Meta-Analysis

**DOI:** 10.3389/fpsyg.2021.798090

**Published:** 2022-01-20

**Authors:** Jonatan Finell, Ellen Sammallahti, Johan Korhonen, Hanna Eklöf, Bert Jonsson

**Affiliations:** ^1^Department of Applied Educational Science, Umeå University, Umeå, Sweden; ^2^Faculty of Education and Welfare Studies, Åbo Akademi University, Vaasa, Finland

**Keywords:** math anxiety, math performance, meta-analysis, working memory, Attentional Control Theory (ACT)

## Abstract

It is well established that math anxiety has a negative relationship with math performance (MP). A few theories have provided explanations for this relationship. One of them, the Attentional Control Theory (ACT), suggests that anxiety can negatively impact the attentional control system and increase one's attention to threat-related stimuli. Within the ACT framework, the math anxiety (MA)—working memory (WM) relationship is argued to be critical for math performance. The present meta-analyses provides insights into the mechanisms of the MA—MP relation and the mediating role of WM. Through database searches with pre-determined search strings, 1,346 unique articles were identified. After excluding non-relevant studies, data from 57 studies and 150 effect sizes were used for investigating the MA—MP correlation using a random-effects model. This resulted in a mean correlation of *r* = −0.168. The database search of WM as a mediator for the MA—MP relation revealed 15 effects sizes leading to a descriptive rather than a generalizable statistic, with a mean indirect effect size of −0.092. Overall, the results confirm the ACT theory, WM does play a significant role in the MA—MP relationship.

## Introduction

It is well established that there is a negative relationship between math anxiety (MA) and math performance (MP; Namkung et al., 2019). There are a few theories that explain how MA affects MP. One that has gained a steady foothold in the literature is the Attentional Control Theory (ACT; Eysenck et al., 2007), which stipulates that anxiety can deplete cognitive resources, which is vital for computing math-related problems. Unexpectedly, there are no systematic literature studies that have explicitly looked at (I) the relationship between MA and WM, and (II) the mediating effect of working memory (WM) on the relationship of MA predicting MP, despite the ACT being the dominant theory. In line with the ACT, the literature points out that MA can deprive WM resources that are needed for complex math computation (see Ashcraft and Krause, 2007 for an overview). This systematic literature study and meta-analysis address these research gaps by synthesizing research that has studied the MA—WM link and WM's mediating effect on the MA—MP relationship.

### Math Anxiety, Working Memory and Performance

MA, which has been of concern for social science researchers since at least the 1950's (Dreger and Aiken, 1957), is commonly defined as feelings of fear, apprehension and tension that interfere when performing math-related activities (Ashcraft, 2002). Math is the single most strenuous subject in the school curriculum which can cause emotions comparable to phobia (Ashcraft and Ridley, 2005), and has been shown to correlate with other types of anxieties, such as test anxiety (Kazelskis et al., 2000; Ashcraft, 2002). Although the two constructs have in some cases shown to correlate strongly with each other, it has been possible to distinguish one from the other through confirmatory factor analysis (Kazelskis et al., 2000).

Ramirez et al. (2018) suggested three potential explanations for MA. The first is framed as the *deficit theory* in which poor math skills explain MA. Ma and Xu (2004) conducted a longitudinal study and found that lower math achievement predicted higher MA. The second is framed as a genetic predisposition. Wang et al. (2014) studied a sample of 514 twin-siblings (*m* = 12.25 years), and their results suggested that ~40% of the variation in MA is due to genetic predispositions, the variation left is caused by environmental factors specific to the child. The third is framed as socio-environmental factors, arguing that children in the lower grades can inherit some of their parents MA, though only if their MA parents reported to frequently support their child (Maloney et al., 2015). On the other hand, Vukovic et al. (2013b) reported that parental involvement in their child's learning significantly reduced MA, which led to an increase in MP (the phenomena was observed in algebraic and word problems, not whole arithmetic problems).

Ashcraft and Moore (2009) propose a risk-factor model of MA which consists of (I) deficits in MP (deficit theory), (II) lack of motivation and (III) weak WM. WM becomes a more relevant factor once the child faces more complex math than single digit arithmetical tasks. Although the developmental aspect of MA is still in need of more research, especially in younger participants to better understand the onset and progression of MA, some studies have found early indications of MA. For example, Mononen et al. (2021) found that MA growth was negatively related to growth in MP in participants as young as 6-year-olds. Another perspective is to what extent MA can arise as soon as formal school is introduced (Maloney and Beilock, 2012). Ashcraft and Moore's (Ashcraft and Moore, 2009) review suggests that MA strengthens in middle school, and peaks around grade 9 or 10.

Concerning the MA—MP link, two diametrically opposite models have been researched in attempts to identify the causality between the two variables (Carey et al., 2016). (I) The Deficit Theory, assuming that poor MP causes MA, was supported in Passolunghi's (2011) study, where children with math learning disabilities exhibited higher MA. In a longitudinal study of Finnish students in grade 3–5, Sorvo et al. (2019) found that arithmetic achievement predicted MA one year later, MA on the other hand, didn't predict later math achievement. The opposing theory, (II) the Debilitating Anxiety Model implies that MA leads to poorer MP. These models combined suggest a reciprocal model (Carey et al., 2016). Other longitudinal studies have provided evidence of reciprocal effects, (Ma and Xu, 2004; Gunderson et al., 2018). Although the relationship between MA and MP may be of a reciprocal nature, in the current study we approached this relationship from the Debilitating Anxiety model by adopting the ACT. Further research is necessary to better understand the MA—MP relation, potentially by addressing influences of individual differences. For instance, Chang and Beilock (2016) discuss the reciprocal relationship between MA and MP and whether individual differences can explain the MA—MP relationship, such as (a) variations in individual cognitive, affective, and motivational factors and (b) environmental factors that consist of teachers and parental MA, and student's perceived classroom environment. In the present study, we focus on WM, a subconstruct of cognition. We use our WM to retrieve the information needed to solve math tasks, keep relevant information about the salient problem, and inhibit irrelevant information.

According to Baddeley and Hitch's (1974) definition of WM, the construct comprises an attentional control system (the central executive), a modality-free processor able to monitor, plan, manipulate information, and select strategies to complete the tasks at hand. This control system is accompanied by two sub-systems: the visuospatial sketchpad and the phonological loop. The WM model has proven to be long-lasting and is referenced in a wide range of research areas (Baddeley, 2010). An additional component was subsequently proposed, namely the episodic buffer, which supposedly is a passive, multimodal storage system that integrates with the subsidiary systems as well as the long-term memory in holding episodic information (Baddeley, 2000). Working memory has consistently been shown to predict math performance (Swanson and Kim, 2007; De Smedt et al., 2009; Wiklund-Hörnqvist et al., 2016). If the math task induces anxiety, the ability of our working memory to maintain information online, and store and retrieve information from long-term memory will be reduced (Ashcraft and Kirk, 2001; Ramirez et al., 2013). Hence, the cognitive processing associated with the to-be-solved mathematical task in combination with math anxiety can overload the working memory system which, consequently, will reduce one's MP. This fits well with the ACT proposition, though one important detail that is vital to the ACT is that anxiety affects processing efficiency (effort spent on a task in relation to performance effectiveness) to a further extent than performance effectiveness (quality of performance; Eysenck et al., 2007). According to the ACT, anxiety impairs attentional processes relevant to the WM, and thereafter redistributing attentional resources on either internal or external stimuli. Internal consisting of worrying thoughts, external of irrelevant distractors threatening to the on-going task.

When reviewing the literature of the MA—WM relationship, the findings varied considerably. Whereas some studies found the MA—WM correlation to be 0.079 and 0.081, respectively (Ching, 2017; Pappas et al., 2019), others found the correlation to be −0.43 and −0.4, respectively (Witt, 2012; Soltanlou et al., 2019). Moreover, in Ashcraft and Kirk's (2001) experiments, the authors found that their subjects WM-scores significantly declined as MA increased, though only in WM-tests containing numerical information, not in language-based tests (*r*_letter−span_ = −0.2; *r*_computation−span_ = −0.4). This is certainly interesting in respect of the utilization of measurement instruments for analyzing the MA—WM link. These differences in effect sizes may also vary as a function of age.

There have been gender differences reported, though to what degree gender can moderate relationships of MA—MP or MA—WM is unclear. Females have displayed higher MA than their male counterparts (Hembree, 1990; Hopko, 2003). In longitudinal studies, Geary et al. (2019) found that MP predicted future MA in females but not males, and in contrast Ma and Xu (2004) found that MP predicted higher MA in males compared to females. Regarding WM, there have been reports of males scoring better at visuospatial measures but not verbal (Robert and Savoie, 2006). Further, Maloney et al. (2012) found that spatial ability mediated the gender—MA relationship.

### Previous Meta-Analyses

Hembree (1990) conducted one of the first, if not the first, meta-analysis on the correlation between MA and MP (high school students *r* = −0.34; college students *r* = −0.31). Later, Ma (1999) synthesized correlations from 26 studies focusing on samples from elementary and high schools (*r* = −0.27). More recently, Namkung et al. (2019), Zhang et al. (2019) and Barroso et al. (2021) pooled correlations of MA and math performance from 223, 131 and 49 studies and found moderate negative correlations (*r* = −0.28; *r* = −0.34; *r* = −0.3). Altogether research has established a robust negative relationship between MA and MP. This is especially of concern as Fan et al. (2019) found, through latent profile analysis on PISA-data, that 22% of US and Korean students and 10% of Finnish students belonged to a high MA profile. Peng et al. (2016) conducted a meta-analysis on the WM—MP relationship from 110 studies and found a moderate correlation (*r* = 0.35). However, the correlation for typically math performing participants was *r* = 0.34, while participants with math difficulties and other cognitive disorders displayed a correlation of *r* = 0.52. Regarding the WM domains, the use of composite measures of WM had a larger correlation with MP (*r* = 0.38) than isolated WM-domains alone (verbal WM: *r* = 30; visuospatial WM: *r* = 0.31), most likely because these tasks include computational requirements which are predictive of MP, both in the visuospatial and the phonological domains (Swanson and Kim, 2007). Moreover Liang et al. (2021) showed that children in the first grade depended more on visuospatial ability than verbal WM, while fifth graders relied on both WM domains in MP situations. These results highlight the progressive and domain specific aspects of WM's influence on MP.

### The Current Study

Though many studies have researched the MA—MP relation, there are still gaps that need to be investigated to better understand the relationship between the two variables. Following the tenets of the ACT, the available executive function resources are depleted as a consequence of MA, and fewer resources are left to the designated task. Lowered WM capacity and diminished executive control following MA will affect performance. For anxious individuals, a worrying stimulus acts as a distractor by reducing the cognitive resources required for successful performance. Conversely, higher WM capacity can act as a protective factor. Although these theories have support from empirical studies, there would be a lot to gain from synthesizing the existing research in order to present a mean correlation. With the ACT setting as the basic framework, we posed two research questions (RQ), assessing the strength of the association between MA and WM and potential mediating effect of WM: (RQ1) what is the mean correlation between MA and WM? (RQ2) what is the mean indirect effect between MA and MP while accounting for WM as a mediator?

## Methods

### Literature Search

The following databases were employed for our search task: Web of Science, Google Scholar, APAPsycNet, Scopus, ProQuest, and the meta-database EBSCO(host)^1^. No restrictions were applied on the dates for when the research-articles were published (earliest possible date until 25th October 2020). A search string was developed by looking up synonyms with various thesauruses. After running pilot-searches the final search-string resulted in the following:

      (“math^*^ achievement” OR “math^*^ performance” OR “math^*^ success” OR “math^*^ score” OR “arithmet^*^” OR “calculation” OR “math^*^ ability”)

                                            AND

          (“math^*^ anxiety” OR “math^*^ worry”)

                                            AND

(“working memory” OR “short term memory” OR “spatial” OR “phonological loop” OR “memory span” OR “digit span” OR “cognit^*^”)

The same search string was used in all the aforementioned databases except for Google Scholar as the character limit was exceeded^2^. The search resulted in 1,901 articles which were imported to the reference management tool EndNote x9. Hand-searched references, which gained an additional 8 articles to the collection, were also imported to EndNote. The initial search accumulated 1,909 articles. After duplicates were removed 1,346 unique articles were reviewed on a title and abstract level.

### Study Criteria

**Figure 1:**
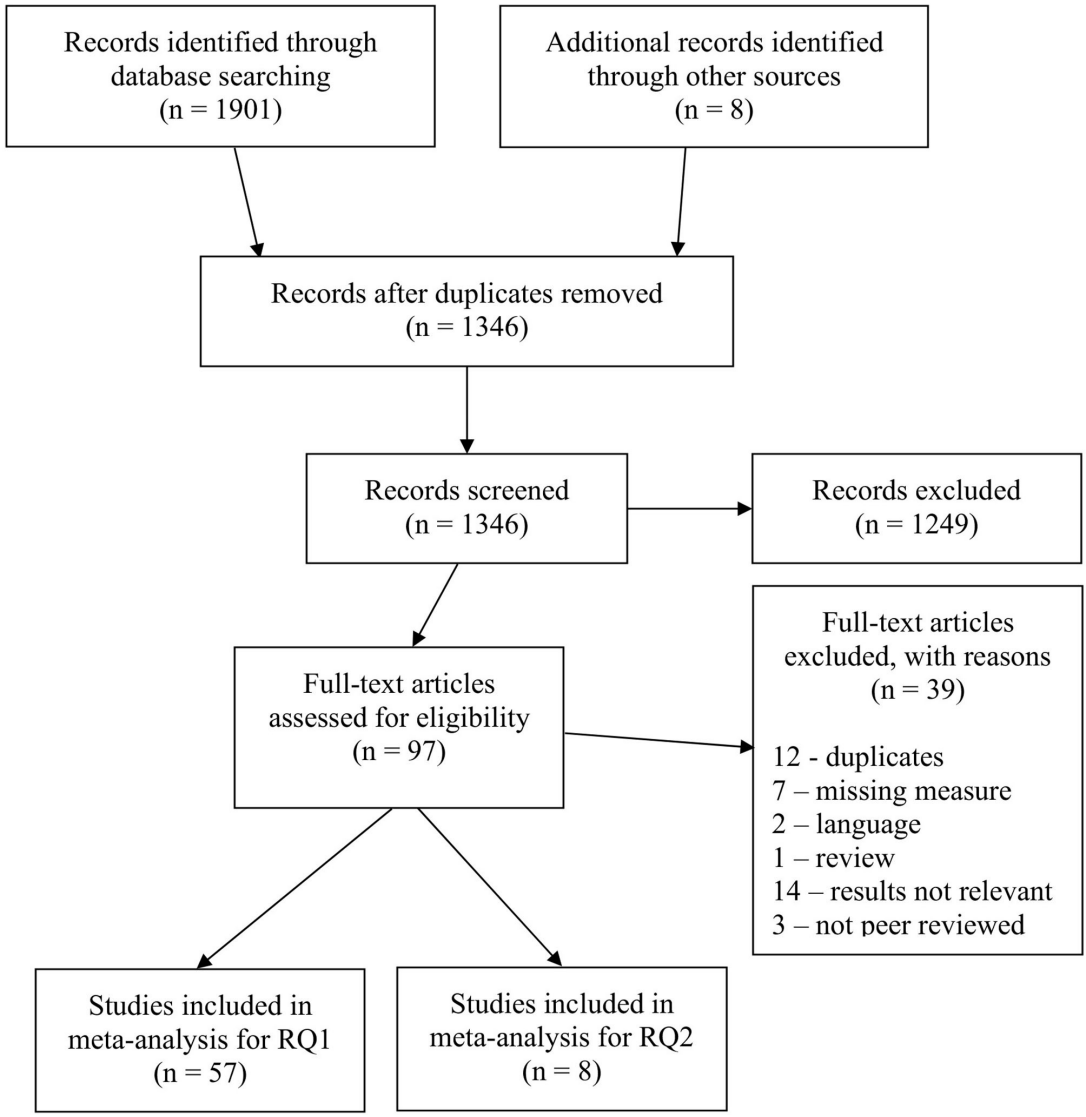
Flowchart of literature search. Based on PRISMA from Moher et al. (2009).

After an initial screening of abstracts, the articles were assessed in full-text. To be included in the full-text analysis, the article had to be peer-reviewed, published, written in English and comprise all three variables of interest: MA, WM, and MP. Further, the results had to include either (or both) a correlation between MA and WM, or a mediation analysis where WM was set to mediate the MA—MP relationship. See Figure 1 for a flowchart of the literature search procedure.

MA is usually conceptualized as two-dimensional, having one affective and one cognitive component (Ho et al., 2000). Regarding MA and the affective dimension, instruments such as MARS (Richardson and Suinn, 1972), different varieties of the MARS, and the AMAS (Hopko et al., 2003) were included in the study. These measurements are designed to capture feelings of nervousness when facing mathematically challenging situations. Regarding MA and the cognitive worry dimension, only the *Faces Pain Scale* instruments (Bieri et al., 1990) have been used in studies of MA (e.g., Trezise and Reeve, 2014a,b). Other measures of anxieties, such as test anxiety, were not considered to meet the MA-criteria and were consequently excluded from the literature review.

Following the Baddeley and Hitch (1974) WM model, the study included measures of the central executive (e.g., operation span), and the sub-systems: phonological loop (e.g., digit span) and visuospatial sketch pad (e.g., Corsi block span). See Table 1 for examples of these measures. Moreover, by extension a categorization into one of the four categories: (1) visuospatial, (2) phonological, (3) central executive + phonological, or (4) central executive + visuospatial was conducted. To qualify into categories one and two, the test must measure verbal or visual memory without applying strains on the central executive. To qualify for categories three or four, the test should require the participants to utilize either spatial or phonological abilities, and simultaneously manipulate information or steer their attention to a second, concurrent task.

**Table 1:** Examples of the most common instruments measuring working memory.

**Component of WM**	**Name**	**Description**	**References**
Phonological	Digit span forward	Participants read back increasingly longer sequences of numbers in the same order as the examiner presented.	Imbo and Vandierendonck, 2007; Buelow and Frakey, 2013; Ramirez et al., 2013; Ashkenazi and Danan, 2017; Skagerlund et al., 2019; Geary et al., 2020
Visuo-spatial	Corsi block test	A sequence of blocks in a quadrant (consisting of blocks) are shown to the participant who later must reproduce the same sequence in the right order.	Trezise and Reeve, 2014a,b; Guthrie and Vallee-Tourangeau, 2018; Lauer et al., 2018; Trezise and Reeve, 2018; Soltanlou et al., 2019; Wang et al., 2020
Central executive + phonological	Operation SPAN	Requires participants to hold information while performing concurrent arithmetic calculations.	Hoffman, 2010; Novak and Tassell, 2015; Novak and Tassell, 2017; Juniati and Budayasa, 2020
	Digit span backwards	Participants read back increasingly longer sequences of numbers in the reverse order of what the examiner presented.	Alamolhodaei, 2009; Alikamar et al., 2013; Georges et al., 2016; Braham and Libertus, 2018; Passolunghi et al., 2019
Central executive + visuo-spatial	Mental rotation	The task is to decide if a given 3D figure is identical or a mirror image of the displayed alternative answers.	Casey et al., 1997; Delgado and Prieto, 2008; Hart et al., 2017; Likhanov et al., 2017; Lauer et al., 2018; Sokolowski et al., 2019
	Corsi block backwards	Participants reproduce a pattern of blocks in a quadrant in the reverse order.	Ashkenazi and Danan, 2017; Soltanlou et al., 2019

*WM, working memory*.

MP was essentially measured in every article that measured MA and WM, these tests differed somewhat. The majority of the studies used standardized math tests, such as the quantitative reasoning ability from the Woodcock-Johnson III-battery (e.g., Miller and Bichsel, 2004). Other tests were researcher's self-designed math test (e.g., Novak and Tassell, 2017) or ordinary class exams (e.g., Alamolhodaei, 2009). Working memory has been shown to predict a broad range of math outcomes, even when other cognitive factors are controlled for (see Raghubar et al., 2010 for an overview), However task differences have been observed. When comparing types of math skill with WM, Peng et al.'s (2016) meta-analysis revealed that word-problem solving and whole-number calculations correlated the strongest with WM (*r* = 0.37; *r* = 0.35), while geometry differed significantly from the aforementioned, displaying a weaker correlation with WM (*r* = 0.23).

### Coding and Interrater Reliability

To measure the reliability of the screening process, the first author read all abstracts and the second author read 40% of the abstracts, this allowed us to calculate a reliability statistic. The Cohen's kappa, an interrater reliability measure, was calculated with the following formula *k* = (*P*_*o*_−*P*_*e*_)/(1−*P*_*e*_) and resulted in an adequate reliability *k* = 0.83 (Cooper et al., 2019). Any disagreements were revisited and discussed by both authors until consensus was achieved. From the abstract screening process, 1,249 articles were excluded leaving 97 articles eligible for full-text assessment.

The first and second author assessed all 97 articles in full text, if articles didn't present relevant results they were excluded. The reliability coefficient for the second comparison was *k* = 0.79. Similar to the first comparison, both authors revisited the articles that were subject for disagreements, in order to discuss and resolve any ambiguities. The first and second authors extracted and independently double coded all of the study's variables, this was in accordance with Cooper et al.'s (2019) recommendations. The study information included variables, such as sample size, sample characteristics, effect sizes, measurement instruments and country, which were all coded into an Excel workbook. Sample size was used to calculate study weights and variances (within study variance) for the effect sizes. Sample size was also needed to calculate the tau-squared (τ^2^), which functioned as a between study variance-measure (Borenstein et al., 2009). All the coded information was compared between the two authors, and any inconsistencies were revisited and discussed. If an inconsistency remained uncertain it was brought up under meetings and assessed by all five authors.

### Analysis

The correlation measure Pearson's *r* for MA and WM was used for the meta-analysis in RQ1. The correlations were transformed into Fisher's 𝓏 effect sizes and variances. As for studies that reported multiple effect sizes based on the same sample, a mean effect size and variance was calculated for the dependent effect sizes. The dependent effects were correlated and estimated with the robust variance estimation (RVE) method with the R-package Robumeta (Fisher and Tipton, 2015). A problem with treating dependent effect sizes that are positively correlated with each other as independent effects, is that the analysis can overestimate the precision and underestimate the error of the mean effect (Borenstein et al., 2009). With this in mind, a mean effect size for dependent effects was computed. The variance for each mean effect size considered the within-study correlation between the outcomes (ρ). Depending on how ρ is specified it can affect the estimation of the between-study variance (τ^2^), the mean error and the actual effect size. The ρ was specified to 0.8 as it was expected that the effect sizes would be correlated with each other. A sensitivity analysis revealed that the analysis was robust over the whole range of estimates of ρ (0–1).

Sub-group analyses (on age, school level, WM domain, verbal- and numerical tests) were performed with the Meta-package in R (Schwarzer, 2007) in accordance with Harrer et al.'s (2021) guide. In the sub-group analysis, moderator variables with multiple categories were transformed into dummy variables, dichotomous variables didn't require transformation. In the sub-group analysis, each effect size was treated as an independent effect.

**Figure 2:**
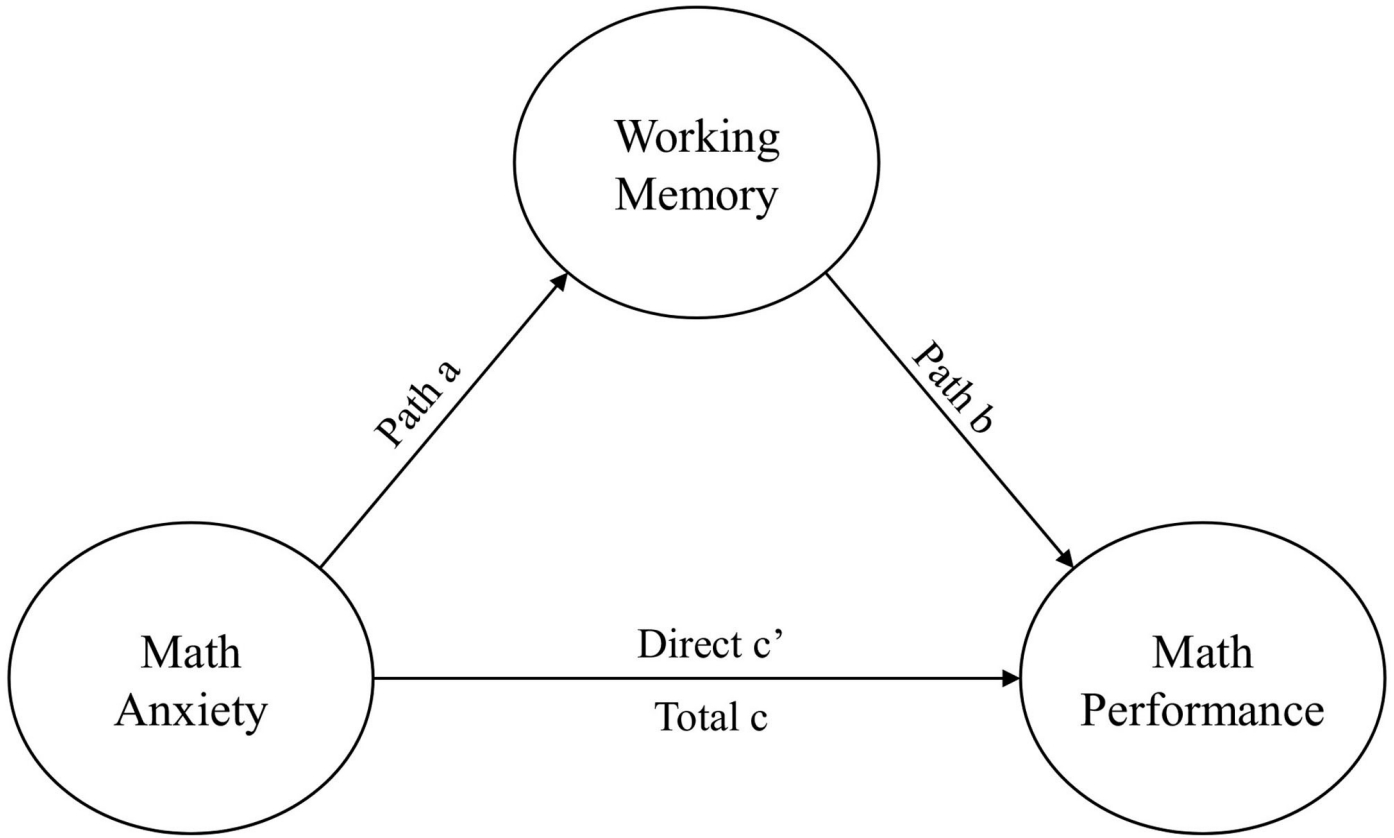
Mediation model.

Regarding the second aim for this study, whether working memory mediates the relationship between math anxiety and math performance, standardized regression coefficients from such models were extracted if reported. In some cases, only unstandardized coefficients were reported. We then used reported descriptive information of means and standard deviations to transform the statistics into standardized coefficients. The main analysis was to compute a summary effect with confidence intervals of the indirect effect, which was calculated by multiplying path a by path b (see Figure 2). Some studies answering RQ2 reported multiple effect sizes. As in RQ1, the RVE method was employed to balance the weights of the studies containing multiple effects. ρ was specified to 0.8. A sensitivity analysis showed that the estimated mean effect and the τ^2^ was slightly affected over the span of 0–1, though within reasonable limits.

The RVE used employed a random-effects model. The rationales for using random-effects model rather than fixed-effect model for answering RQ1 were: (I) the samples originated from different populations as samples varied by age, country, and other factors. (II) A test of heterogeneity was significant (*p* < 0.001) and the *I*^2^-index (*I*^2^ = total heterogeneity/total observed variability), a statistic that can offer an indication of the degree of heterogeneity, resulted in 75.6% heterogeneity, which can be considered a high value (Higgins et al., 2003). Regarding the analysis for RQ2, there was evidence of some heterogeneity (*I*^2^ = 47 %) in addition to the varied samples. This also suggested that a random-effects model would be appropriate.

Potential bias in our data was analyzed by fitting a funnel plot (**Figure 4**) to the data for subsequent visual inspection. Egger's regression test was carried out for assessing asymmetry in the funnel plot. The inverse of the standard errors was applied to the vertical axis as it's usually the recommended practice for bias-detection in meta-analysis (Sterne and Egger, 2001). This brings studies with larger sample sizes to the top of the funnel and smaller closer to the bottom.

### Sub-Group Analysis

The fairly high level of heterogeneity gave reason to investigate whether certain variables affected the MA—WM relationship. The sub-groups that were chosen were based on sample characteristics and the large amount and diversity of the WM measures. These consisted of age, school-level, WM-domain, and type of WM-test. Age was categorized into participants (I) <12 years, (II) ≥12 years, (III) samples consisting of participants around the age 12. The decision for our cut-off value of 12-years for the age-variable, was based partly on earlier research that has found MA to increase in the early teens (Ashcraft and Moore, 2009), and partly on a related earlier meta-analysis' age grouping (Caviola et al., 2017). School level consisted of (I) primary school, (II) high school, (III) university. WM-domains consisted of: (I) visuospatial, (II) phonological, (III) visuospatial + central executive and (IV) phonological + central executive. WM-tests were divided into (I) verbally based and (II) tests without verbal elements. WM-tests were also divided based on their numerical attributes into either (I) numerical tests, or (II) non-numerical tests.

## Results

### Mean Correlation Working Memory—Math Anxiety

To answer RQ1 effect sizes were pooled from a total of 57 articles, 66 unique samples comprising of 16,589 participants and 150 correlation coefficients. The mean correlation between MA and WM was −0.168 with confidence intervals on the 95% level ranging from −0.203 to −0.133 (see Table 2). The sub-group analysis of age, school level, and numerical based WM-tests were all significant, *p* < 0.001, *p* < 0.01, and *p* < 0.05, respectively.

**Table 2:** Mean correlation and sub-group analysis on the MA—WM relationship.

**Subcategory**	**Effect sizes**	**r**	**95 % CI**.	**τ^2^**	**Between group statistics**	
					**Q**	***p*-value**
Mean correlation^a^	150	−0.168	[−0.203; −0.133]	0.0123		
Age					23.16	<0.0001
Child <12 year	29	−0.101	[−0.148; −0.053]	0.0098		
Child ≤ > 12 year	29	−0.126	[−0.161; −0.090]	0.0044		
Child ≥ 12 year	92	−0.219	[−0.251; −0.188]	0.0166		
School level					14.02	0.0029
Primary	42	−0.113	[−0.150; −0.075]	0.0085		
High school	39	−0.205	[−0.257; −0.153]	0.0235		
University	64	−0.202	[−0.238; −0.165]	0.0134		
WM category					9.27	0.0547
Visuospatial	33	−0.185	[−0.228; −0.142]	0.0103		
Phonological	20	−0.125	[−0.162; −0.087]	0.0015		
Phono + CE	63	−0.204	[−0.247; −0.161]	0.0218		
Visuo + CE	31	−0.167	[−0.215; −0.118]	0.0134		
Verbal WM-test					1.85	0.1738
Verbal based test	80	−0.192	[−0.224; −0.159]	0.0161		
No Verbal elements	70	−0.159	[−0.193; −0.124]	0.0139		
Numerical based test					5.91	0.0151
Numeric	64	−0.212	[−0.251; −0.173]	0.0177		
Non-numeric	86	−0.152	[−0.181; −0.123]	0.0123		

a *Estimated with robust variance estimation method in a random effects model, standard error = 0.0181. WM, working memory; MA, math anxiety; CE, central executive*.

### Participant Age

Age was one of the clearest moderating variables. Participants of under the age of 12 had a weak MA—WM correlation (*r* = −0.101, 95 % C.I. [−0.148; −0.053]). Participants around the age of 12 (samples that ranged from under 12 to over 12), had a slightly stronger MA—WM relationship (*r* = −0.126, 95 % C.I. [−0.161; −0.090]). Participants over the age of 12 exhibited the strongest MA—WM relationship (*r* = −0.219, 95 % C.I. [−0.251; −0.188]) which was significantly stronger than the two other age groups (*p* < 0.001). A meta-regression analysis was carried out with participant mean age as the predictor of the relationship. This resulted in a small, significant effect (β = −0.005, std. error = 0.002, *p* < 0.01). Further, the MA—WM correlation increased in strength from primary to high-school (*r* = −0.113; *r* = −0.205). The correlation didn't significantly differ from high school to university (*r* = −0.205; *r* = −0.202).

Regarding the gender aspect, there was not enough available data for investigating differences between males and females (number of effect sizes: males = 2; females = 12) in the MA—WM relation. This would be of interest for future research as previous research has shown that females experience higher levels of MA compared to males (Hembree, 1990).

### Working Memory Category and Tests

A between-group analysis revealed a marginal main effect of WM categories (*p* = 0.055). Descriptively, this effect can be seen in Table 2, represented by almost non-overlapping confidence intervals. Hence, the phonological (*r* = −0.125, 95 % C.I. [−0.162; −0.087]) and phonological + central executive domain's (*r* = −0.205, 95 % C.I. [−0.247; −0.161]) confidence intervals (95%) overlapped with only one thousandths decimal point. A meta-regression analysis revealed that phonological domain predicted the MA—WM correlation (β = 0.064, std. error = 0.036, *p* < 0.05). No other WM category revealed significant effects, neither sub-group analysis nor meta-regression analysis. The analysis of verbal WM tests revealed no main effect while the analysis of numerical tests revealed a main effect (*p* < 0.05), with a stronger MA—WM correlation for numerical tests (*r* = −0.212) compared to non-numerical tests (*r* = −0.152). See Table 2 for details.

### Mean Correlation of Indirect Effect

As for RQ2, 10 studies reportedly measured the mediation model of interest (see Figure 2). However, one study didn't report the necessary statistics for the analysis, as their aim was more focused on the gender aspect, though they did mention that MA failed to show an indirect or a direct effect on MP in a model accounting for WM, other parallel mediators, and covariates. A second study was dropped because the reported statistics were out of proportion and couldn't be transformed into standardized values. Though in the same study, the authors reported a significant indirect effect of MA predicting MP while accounting for WM. A sample size of 1,824 participants from eight studies with a total of 15 effect sizes of the indirect effects of MA predicting MP while accounting for WM as a mediator, were synthesized with the RVE method and resulted in a significant negative indirect effect (*r* = −0.092, *p* < 0.05) between MA predicting MP while accounting for WM. See Table 3 for details.

**Table 3:** Mean correlation of the indirect effect.

**Studies**	**Effect sizes**	**Estimate**	**Std. Error**	**95 % C.I**.	**τ^2^**
8	15	−0.0923	0.0326	[−0.169; −0.0152]	0.00426

*Indirect effect of math anxiety predicting math performance while accounting for working memory as a mediator*.

### Funnel Plot Analysis

**Figure 3:**
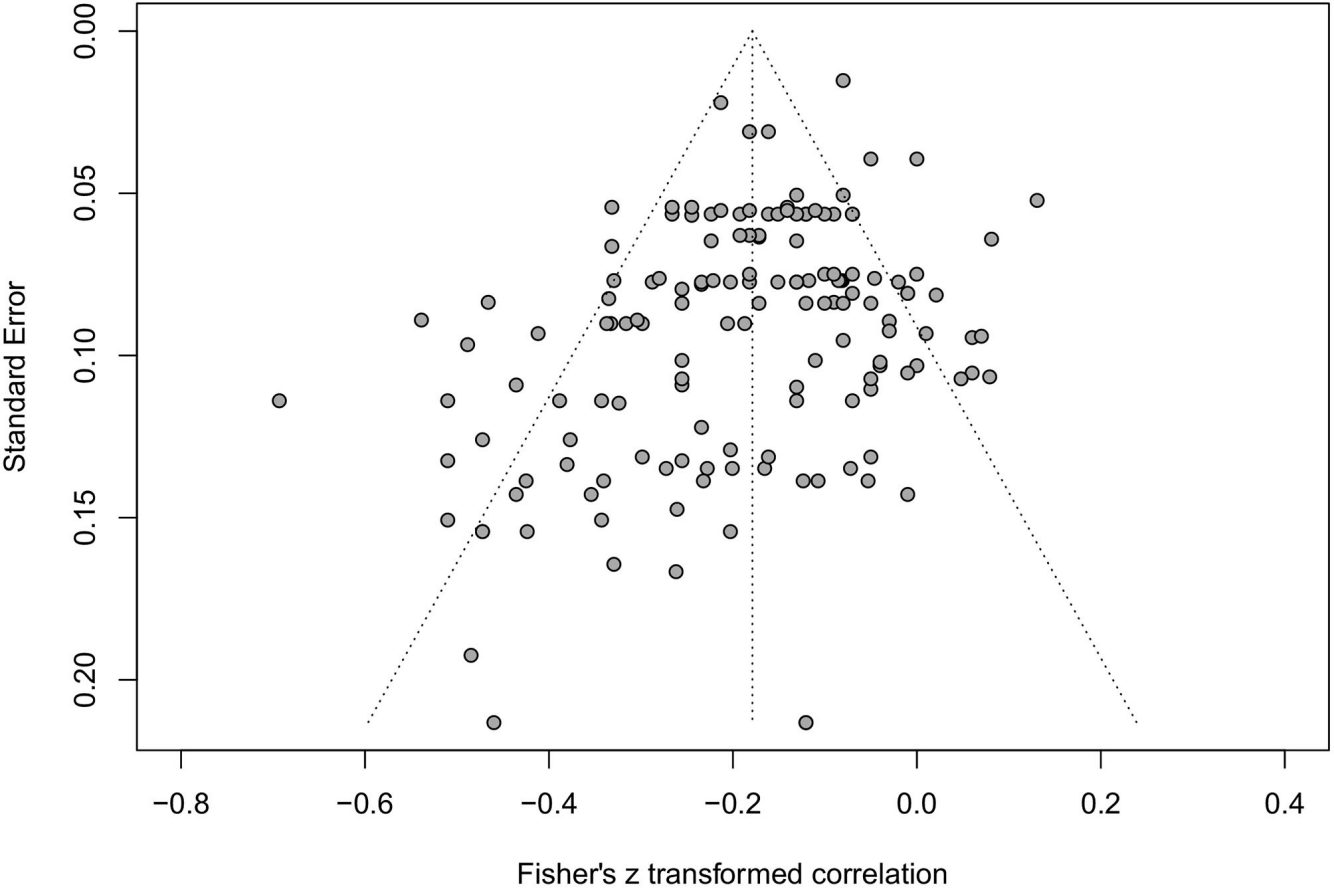
Funnel plot of the math anxiety—working memory effects.

A funnel plot with study effect sizes for RQ1 was analyzed (see Figure 3). Visual inspection of the effect sizes in relation to the funnel suggested the data wasn't normally distributed as effect sizes occurred outside the funnel. Egger's regression test for asymmetry was significant (*p* < 0.001) confirming the asymmetric data. It's possible that the data suffers from small-study effects as stronger effects are seen in smaller studies with larger standard errors (Rücker et al., 2011). A bias-corrected analysis in the form of a trim-and-fill method can function as a sensitivity analysis (Peters et al., 2007). Adding 27 effect sizes, to mirror the extremes, into the funnel plot (*n* = 173) lowered the average correlation to −0.136, while remaining significant (95 % C.I. −0.164 to −0.108, *p* < 0.001). A meta-regression was performed on publication year which suggested that the effect slightly decreases with more recent publications (β = 0.008, *SE* = 0.003, *p* < 0.001).

## Discussion

This meta-analysis investigated the relationship between MA and WM, and the mediating effect of WM on the relationship of MA predicting MP. The results based on 66 unique samples showed that MA had a significant negative correlation with WM (*r* = −0.168), which according to Hemphill (2003), is interpreted as a small effect size. This relationship varied significantly as a function of age, school level, WM category and whether WM tests were numerically based or not. Furthermore, a significant indirect effect (−0.092) of MA on MP through WM was found based on eight unique samples. There has been plenty of research reporting the MA—WM statistic, though these effect sizes have varied from study to study. A synthesized correlation of MA—WM has been reported in Namkung et al. (2019) which contrasts our study as they found a non-significant correlation between the two variables MA and WM (*r* = −0.08), though their study-focus was on the MA—MP correlation. The current study presents a mean correlation on the MA—WM link, based on synthesized effect sizes from published, peer reviewed research.

### Participant Characteristics

There was a clear age effect in the MA—WM relationship, as older participants displayed a stronger negative MA—WM relationship. This was also mirrored in the school-level analyses. From primary school to high school the MA—WM relationship grew stronger, leveling off in high school and remained static throughout university. These results are in line with Ashcraft and Moore's (Ashcraft and Moore, 2009) suggestion that MA peaks around grade 10, and levels off shortly after. Moreover, the age-effect in the MA—WM correlation in the present study and the MA—MP correlation in Zhang et al.'s (2019) meta-analysis is up to high school level very similar. With the exception that University students displayed a lower MA—MP correlation than high school students, in their study.

### Working Memory

The WM measures selected were based on Baddeley and Hitch's (1974) model and thus were categorized into phonological, visuospatial or combinations of the central executive and the phonological or visuospatial sub-systems. Between-group analysis, between phonological—MA and phonological + centra executive—MA, revealed a marginal significant main effect (*p* = 0.055; Table 2), as indicated by almost separated confidence intervals (95%). This in combination with the meta-regression analysis showing that the phonological domain predicted the relationship of MA—WM, indicate that the phonological domain might differ from the rest. The phonological + central executive measures correlated more strongly with MA (*r* = −0.205) compared to tests measuring solely the phonological loop (*r* = −0.125). These results are interesting from the perspective of the ACT, which theorizes that anxiety functions as a distractor that depletes cognitive resources required for cognitively demanding problems. Just like its predecessor, the processing efficiency theory (Eysenck and Calvo, 1992), the ACT emphasizes that anxiety has a greater impact on the executive component of working memory. The interpretation of our results can to some extent support the idea, that anxiety affects short term memory storage and attentional processes to a greater extent compared to short term memory storage alone. This is however not applied to our results of the visuospatial domain as most of the C.I. in visuospatial and visuospatial + central executive overlapped, indicating that the correlation was fairly similar.

### Working Memory as a Mediator

The debilitating anxiety model suggests that anxiety can cause deficiencies in math performance. Deficits in MP caused by MA can fully or at least partly be mediated by cognitive processes. Indeed, our results showing a significant negative indirect effect of MA on MP via WM, support the ACT. When one experiences worrying thoughts, working memory resources are spent on irrelevant stimuli, thus, limiting the processing capacity required for performing the math problems at hand. Regarding RQ1 our sub-group analysis on numerical-based WM-tests supports Ashcraft and Kirk's (2001) findings that MA correlates more strongly with WM if the WM-measures are numerical. This implies that numbers *per se* can trigger anxiety to large extent and thus deplete cognitive processing resources. But why is higher MA significantly associated with lower WM, when looking at non-numerical WM measures? An explanation could potentially be found in the lack of construct independence. Although MA is defined as a feeling of nervousness in a math-related context (Ashcraft, 2002), its relative high correlation with test anxiety (Kazelskis et al., 2000), indicates associations to other forms of anxieties. Indeed, studies have found that MA relates moderately to strongly with state anxiety, (*r* = 0.30, Hopko, 2003), general anxiety (*r* = 0.5, Llabre and Suarez, 1985) and test anxiety (*r* = 0.687, O'Leary et al., 2017).

### Limitation

**Figure 4:**
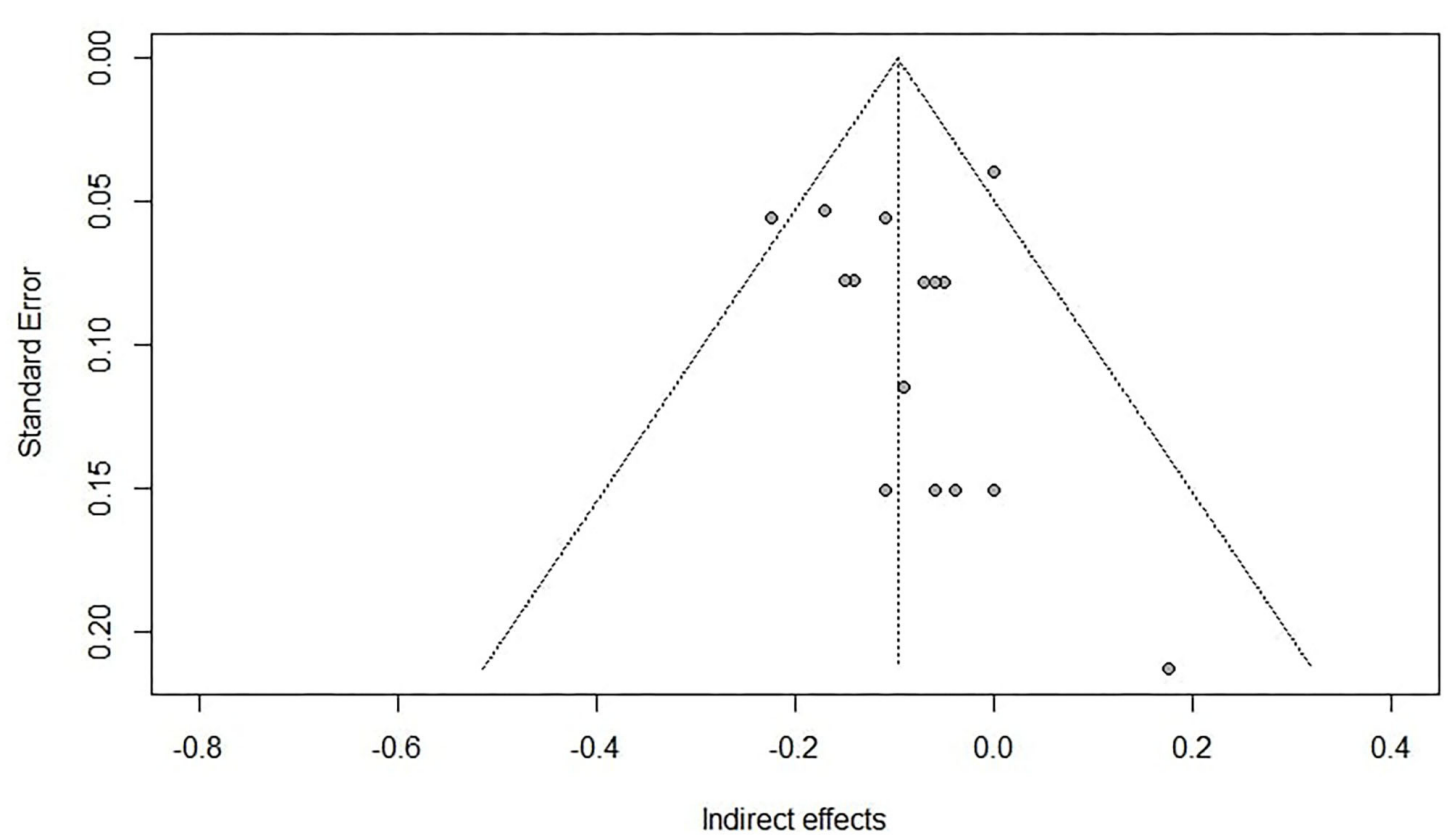
Funnel plot of indirect effects.

The results from the funnel plot (Figure 4) with some studies outside the funnel in combination with Egger's regression test for asymmetry and the trim-and-fill method that lowered the average correlation indicated a publication bias. Especially as the current study only collected peer-reviewed, published studies, which can be seen as a limitation as some research (e.g., theses) will be left out. However, the effect sizes collected for our meta-analysis were seldom the main aim in the included literature. These effect sizes were most often found in sections where descriptive information or background variables were presented, typically not being an object of publication bias- providing a counterargument for publication bias. Moreover, a meta-regression revealed that publication date predicted the MA—WM relationship, which partly could explain the asymmetry in the funnel plot. With respect to the heterogeneity that characterizes the data (Higgins et al., 2003) we opted for implementing a random-effects model for RQ1. For instance, the data involves a vast and diverse measurement for both MA and WM, and the results show evidence of how the relationship increases in strength as the population grows older (Table 2). Imputing new data for bias-reasons may not be the answer in this case. As the τ^2^ was significant, it is not always good practice to correct for this type of bias as the chances of unreliable results increase (Peters et al., 2007).

Regarding RQ2, an evident problem had to be addressed, namely the small number of studies answering the research question. This challenged the external validity and is considered a limitation. The reader should regard the results for RQ2 as descriptive and not prescriptive.

## Conclusion

This study presents a robust significant negative relationship between MA—WM confirming the relevance of the ACT. A significant indirect effect from MA predicting MP while accounting for WM is also shown, though this relationship should be interpreted with caution as there was a limited number of studies answering this question. Researchers should be aware of what type of WM-measure is used and whether the instrument is of numerical nature or not, which could impact on the MA participants. Further, our results suggest that WM-measures of both the phonological and executive components have a stronger association with MA than phonological storage processes alone, which also is stated in the ACT. We recognize that there is still a shortage of data for determining the precision and certainty of the indirect effect that MA has on MP. This question still requires further investigation. Lastly, there is the age aspect to the MA—WM relationship. Our results indicate that somewhere between primary and high school the relationship develops in strength and levels off in university. This protracted development may be connected to younger children experiencing less MA than older (Wigfield and Meece, 1988; Ashcraft and Moore, 2009). If that is the case, the findings of this study highlight the importance of early interventions to suppress anxieties that can have detrimental effects on math performance and be pertinent to general cognitive processing abilities.

## Data Availability Statement

The original contributions presented in the study are included in the article/supplementary material, further inquiries can be directed to the corresponding author.

## Author Contributions

JK and BJ came up with the idea for the study. JF, ES, JK, HE, and BJ jointly contributed to the study's conceptualization and revised the manuscript for important intellectual content. JF performed the statistical analysis and wrote the first draft of the manuscript. All authors contributed to the manuscript and read and approved the submitted version.

## Funding

Funding was received from the Swedish Research Council, VR, Grant (2019–03928).

## Conflict of Interest

The authors declare that the research was conducted in the absence of any commercial or financial relationships that could be construed as a potential conflict of interest.

## Publisher's Note

All claims expressed in this article are solely those of the authors and do not necessarily represent those of their affiliated organizations, or those of the publisher, the editors and the reviewers. Any product that may be evaluated in this article, or claim that may be made by its manufacturer, is not guaranteed or endorsed by the publisher.

## Footnotes

^1^EBSCO(host) included the following databases: Academic Search Premier, APA Psycinfo, Business Source Premier, CINAHL, ERIC, MEDLINE, SocINDEX and SPORTDiscus.

^2^The following were excluded in Google Scholar: “calculation,” “math^*^ ability,” “memory span” and “digit span”.
